# Computational Identification and Analysis of Ubiquinone-Binding Proteins

**DOI:** 10.3390/cells9020520

**Published:** 2020-02-24

**Authors:** Chang Lu, Wenjie Jiang, Hang Wang, Jinxiu Jiang, Zhiqiang Ma, Han Wang

**Affiliations:** 1 School of Information Science and Technology, Northeast Normal University, Changchun 130117, China; luchang404@hotmail.com (C.L.); Jiangwj057@nenu.edu.cn (W.J.); wangh013@nenu.edu.cn (H.W.); jiangjx551@nenu.edu.cn (J.J.); 2 Institute of Computational Biology, Northeast Normal University, Changchun 130117, China; 3 Department of Computer Science, College of Humanities & Sciences of Northeast Normal University, Changchun 130117, China

**Keywords:** ubiquinone-binding proteins, XGBoost, binding domain motifs, gene ontology, KEGG pathway

## Abstract

Ubiquinone is an important cofactor that plays vital and diverse roles in many biological processes. Ubiquinone-binding proteins (UBPs) are receptor proteins that dock with ubiquinones. Analyzing and identifying UBPs via a computational approach will provide insights into the pathways associated with ubiquinones. In this work, we were the first to propose a UBPs predictor (UBPs-Pred). The optimal feature subset selected from three categories of sequence-derived features was fed into the extreme gradient boosting (XGBoost) classifier, and the parameters of XGBoost were tuned by multi-objective particle swarm optimization (MOPSO). The experimental results over the independent validation demonstrated considerable prediction performance with a Matthews correlation coefficient (MCC) of 0.517. After that, we analyzed the UBPs using bioinformatics methods, including the statistics of the binding domain motifs and protein distribution, as well as an enrichment analysis of the gene ontology (GO) and the Kyoto Encyclopedia of Genes and Genomes (KEGG) pathway.

## 1. Introduction

Ubiquinone, also known as coenzyme Q (CoQ), is a fat-soluble organic compound that is ubiquitous in all cell membranes of all animals and most bacteria. It is a powerful antioxidant in cell membranes and lipoproteins, an important component of the electron transport chain, and plays a central role in the process of mitochondrial oxidative phosphorylation [1,2]. Coenzyme Q-10 (CoQ10) is the most common form of ubiquinones, where Q represents the quinone chemical group, and 10 represents the number of isoprenyl chemical subunits in its tail [3]. CoQ10 tends to concentrate in organs with high energy requirements, such as the heart, liver, and kidney. Special interest in CoQ10 has arisen from the fact that it associates with many types of diseases, such as heart disease [4,5], chronic kidney disease [6,7], and cancer [8,9]. However, the pharmacology of CoQ10 is not entirely clear, and further comprehensive studies are required for clarity.

Like other ligands, ubiquinones perform their functions mainly by binding with receptors. Ubiquinone-binding proteins (UBPs) are receptor proteins that dock with ubiquinones. The identification and characterization of UBPs provide important clues for understanding the metabolic pathways involving ubiquinones. Currently, abundant protein sequence data have been garnered. Recognizing UBPs from large numbers of proteins through traditional biochemical experiments has become more and more difficult because of high time, labor, and financial costs [10,11]. In this case, analyzing and identifying UBPs by computational methods will assist in annotating protein functions, help to characterize motifs, provide insights into the pathways associated with ubiquinones, and contribute to the development of the pharmaceutical industry.

In recent decades, a number of prediction models have been proposed to identify ligand-binding proteins from sequence-derived information using computational methods [12]. For example, Shaherin B. et al. [13] identified growth hormone-binding proteins by using the extremely randomized tree; Chauhan S. et al. [14] predicted DNA-binding proteins using multilayer perceptron (MLP) and deep convolutional neural network (CNN); and Pan X.Y. et al. predicted RNA-binding proteins using CNN and bidirectional long short term memory network (BLSTM) [15]. Although great attention has been paid to ubiquinones and UBPs by biochemical and medical researchers, few studies have been done in the field of bioinformatics and, at present, no computational prediction methods currently exist for UBPs identification.

In this study, we proposed a machine learning-based predictor (UBPs-Pred) to identify UBPs from primary protein sequence information. First, we collected UBPs from the Swiss-Prot database [16] to establish a high-quality benchmark dataset. Then, three kinds of sequence-derived features were extracted, including amino acid composition (AAC), dipeptide composition (DC), and position-specific scoring matrix (PSSM). In order to determine the optimal features needed to provide key clues for UBP identification, random forest (RF) was used to rank the importance of the features, and incremental feature selection (IFS) was used to build the optimal subset of features. Subsequently, the selected features were fed into the extreme gradient boosting (XGBoost) classifier. To fully leverage the advantages of XGBoost, multi-objective particle swarm optimization (MOPSO) was employed for parameter tuning. The results of the independent testing demonstrated that the predictor achieved excellent performance, with a Matthews correlation coefficient (MCC) of 0.517. The source code can be found at https://github.com/NENUBioCompute/UBPs-Pred.

Besides building a UBPs predictor, we analyzed UBPs by using bioinformatics methods. We tried to discover the motifs of ubiquinone-binding domains and found eight motifs that were statistically enriched. Then, the distribution of UBPs was counted, and we determined that most of the UBPs were membrane proteins. The statistics on the distribution of superfamilies showed that the identified UBPs were members of various kinds of superfamilies that were involved in complex biological processes. To further analyze the functions of UBPs, especially in the human body, gene ontology (GO) enrichment and Kyoto Encyclopedia of Genes and Genomes (KEGG) pathway enrichment of human UBPs were analyzed, and we found that four disease pathways appeared in the top 11 significantly enriched KEGG pathways.

## 2. Materials and Methods

### 2.1. Benchmark Datasets

We built benchmark datasets for training and testing the proposed model. Positive samples were obtained by the following procedures. First, 10,224 UBPs were collected from Swiss-Prot (released on June 5, 2019) by parsing the annotation of proteins. Second, we removed the proteins that contained unknown residues or less than 50 residues because unknown residues may confuse the prediction model, and sequences of less than 50 residues tend to be peptides rather than complete protein sequences. Next, to reduce the negative influence of data redundancy and homology bias [17], we removed the homologous proteins with >30% similarity. CD-HIT [18] was used to cluster the remaining proteins with a sequence identity cut-off of 0.3, and the representative protein of each cluster was selected. Finally, we obtained 524 UBPs that were non-redundant positive samples.

Except for the 10,244 UBPs, all other proteins were considered to be non-UBPs. We collected 524 non-redundant negative samples via the same procedure as the positive ones. A total of 450 UBPs and 450 non-UBPs were randomly selected and combined as the training dataset (UBPs900), and the remaining 148 samples (74 UBPs and 74 non-UBPs) were used as the testing dataset (UBPs148). To avoid the potential instability of the predictor, we repeated the process for extracting negative samples five times and built five benchmark datasets. All the predicted results reported in this work were the average of the five experiments using these datasets. The benchmark datasets can be found in the Supplementary Materials (Data S1: BenchmarkDatasets.zip).

### 2.2. Feature Construction

The primary amino acid sequences of the proteins cannot be directly used by machine learning methods. Encoding proteins with digital features is the first step in constructing a predictor. The quality of the features directly affects the performance of the predictor. We chose three types of features to encode the proteins: the amino acid composition (AAC), the dipeptide composition (DC), and the position-specific scoring matrix (PSSM).

#### 2.2.1. Amino Acid Composition (AAC)

The twenty amino acids directly encoded by triplet genetic codons are known as the standard amino acids. A protein sequence is a linear chain of standard amino acid residues. Proteins differ from one another primarily in their arrangement of amino acids, which results in the protein folding into a specific structure that determines its function. The amino acid composition (AAC) represents the distribution of twenty amino acids in proteins, which is the most intuitive way to describe the differences between proteins. Numerous methods have been developed by using AAC as an important component of features for annotation of protein function [19,20]. The amino acid composition of a protein is a 20-dimension vector in which the element is the ratio of the corresponding residue that appears in the protein. For a given protein, the AAC feature can be defined as

(1) $$f_{AAC}\left(i\right)=\begin{array}{cc} \frac{AA_{i}}{L} & i\in \left\{1,\;2,\;3,\ldots ,20\right\} \end{array}$$

where $AA_{i}$ represents the number of the i-th type of amino acids appearing in the protein, and L represents the length of the protein.

#### 2.2.2. Dipeptide Composition (DC)

Similar to the feature of AAC, the dipeptide composition (DC) is another amino acid composition descriptor that introduces the intrinsic information of the protein sequences. The DC feature has been applied in many predictive problems, such as mycobacterial membrane protein type identification [21] and bioluminescent protein prediction [22]. DC is a 400-dimension vector that indicates the occurrence frequency of all the possible adjacent amino acid pairs. The element of the vector is the ratio of the corresponding amino acid pair that appears in the protein. Given a protein, the DC feature can be defined as

(2) $$f_{DC}\left(i,j\right)=\begin{array}{cc} \frac{AA_{i}AA_{j}}{L-1} & i,j\in \left\{1,\;2,\;3,\ldots ,20\right\} \end{array}$$

where $AA_{i}AA_{j}$ represents the number of the corresponding adjacent amino acid pair that appears in the given protein, and L represents the length of the protein.

#### 2.2.3. Position-Specific Scoring Matrix (PSSM)

With the evolutionary process over successive generations, certain heritable characteristics of proteins become more common or rarer within a protein family. The similarities of evolutionary conservation are always associated with structural or functional needs [23,24]. The position-specific scoring matrix (PSSM) is one of the most effective and widely used descriptors that represent the evolutionary conservation of protein sequences. PSSM has received a great deal of attention from researchers and has been successfully used in a number of problems, such as protein secondary structure prediction [25] and DNA-binding protein identification [26,27]. The PSSM of a given protein can be obtained by using the PSI-BLAST [28] tool to search the Swiss-Prot database (released on June 5, 2019) through three iterations, with an E-value threshold of 0.01. The E-value is the statistical measurement of the number of expected matches in the database. The lower the E-value, the more likely the match is to be significant. The PSSM of a protein can be defined as

(3) $$PSSM=\left[\begin{array}{ccc} S_{1,AA_{1}} & \begin{array}{cc} S_{1,AA_{2}} & \ldots \end{array} & S_{1,AA_{20}}\\ S_{2,AA_{1}} & \begin{array}{cc} S_{2,AA_{2}} & \ldots \end{array} & S_{2,AA_{20}}\\ \begin{array}{c} \vdots \\ S_{L,AA_{1}} \end{array} & \begin{array}{cc} \begin{array}{c} \vdots \\ S_{L,AA_{2}} \end{array} & \begin{array}{c} \vdots \\ \ldots \end{array} \end{array} & \begin{array}{c} \vdots \\ S_{L,AA_{20}} \end{array} \end{array}\right]$$

where $S_{i,AA_{j}}$ is the element’s value of PSSM, which represents the occurrence frequency of $AA_{j}$ at the i-th position of the given protein in the result of multiple sequence alignment. L represents the length of the protein. For a given protein, we further flattened the original PSSM into a vector with equal length and obtained a 400-dimension feature that can be defined as

(4) $$f_{PSSM}\left(i,j\right)=\begin{array}{cc} \overset{L}{\underset{i=1}{\sum }}S_{i,AA_{j}}\times \delta \left\{\begin{array}{c} \delta =1,\;R_{i}=AA_{j}\\ \delta =0,\;R_{i}\neq AA_{j} \end{array}\right. & i,j\in \left\{1,\;2,\;3,\ldots ,20\right\} \end{array}$$

where $R_{i}$ represents the i-th residue in the protein sequence.

In summary, 820-dimensional features were used to encode proteins, including 20 AAC, 400 DC, and 400 PSSM.

### 2.3. Feature Selection Strategy

According to the descriptions in the previous section, three categories of features were used for the prediction model: AAC, DC, and PSSM. All of these features were statistically based and considered all amino acid types and pairwise combinations. Although these features introduced the intrinsic information of protein sequences, noisy and redundant features inevitably existed. To better understand these features and correctly verify the prediction model, a feature selection strategy was required. By using the process of feature selection, noisy features were removed, and the prediction performance was further improved.

Random forest (RF) [29,30,31] was used to rank the importance of the features in this work. The importance score for a given feature was computed by averaging the decrease in the Gini index when the feature was chosen to split the nodes. Features with larger scores were ranked as more important [32]. A total of 526 out of 820 features remained in this step because the important scores of the other features were 0. We believed that the more important features would contribute more to the classification performance. According to the ranked feature list, the incremental feature selection (IFS) [33] strategy was used to build a series of feature subsets by increasing the features one by one. For each feature subset, a model was built and evaluated. The model that achieved the highest MCC value was chosen as the final prediction model, and the features in the corresponding subset were chosen as the optimal features. The optimal feature subset contained 242 features, including 9 AAC, 87 DC, and 146 PSSM features.

### 2.4. Binary Prediction Model

Extreme gradient boosting (XGBoost) [34] is a decision tree-based ensemble algorithm that applies the principle of boosting weak learners using gradient descent architecture. XGBoost is an optimization of the gradient boosting algorithm using both software and hardware optimization techniques. XGBoost was first proposed by Chen T.Q. and Guestrin C. in 2016. Since its introduction, this algorithm has become the driving force in several cutting-edge research fields, including (but not limited to) bioinformatics. XGBoost has been widely used in many bioinformatics problems, such as gene expression value prediction [35], protein subcellular localization [36], and internal ribosome entry site prediction [37].

### 2.5. Parameter Tuning

XGBoost is a highly sophisticated algorithm that involves multiple parameters. We needed to consider parameters and tune their values to fully leverage the advantages of XGBoost. These parameters could be divided into three categories: general, booster, and learning task parameters. General parameters define the overall functionality, notable among them are booster and nthread. “booster” controls the type of booster to be run at each iteration, which can be gbtree (tree-based booster, which is the default) or gblinear (linear booster). We set it as default because the tree-based booster significantly outperforms the linear booster. “nthread” controls the number of cores used for parallel processing based on the maximum number of threads that are available. We set nthread as default to obtain the optimal speed. In each iteration, the booster parameters define the individual tree-based booster, and the learning task parameters guide the optimization objective. There are plenty of parameters in both categories, but not all of them are worthy of tuning. We tuned eight parameters that significantly influence the predictor, including learning_rate, n_estimators, max_depth, subsample, colsample_bytree, gamma, reg_alpha, and reg_lambda. The details of these parameters are illustrated in Table 1.

Tuning the parameters of XGBoost is a typical multi-objective optimization problem, and the traditional grid search method is extraordinarily time-consuming. In this case, multi-objective particle swarm optimization (MOPSO) [38] was employed to obtain the ideal values of these parameters. Particle swarm optimization (PSO) [39] is a single-objective optimization algorithm that mimics the social behavior of bird flocks. MOPSO is an extension of PSO for multi-objective optimization problems, which takes the concept of Pareto dominance to list the best solutions to guide the movement of the particles. We randomly initialized 80 sets of 8-dimension vectors as the particle population, in which each item in the vector of a particle represented one parameter of XGBoost. The stopping criterion was when the maximum number of iterations was reached (200 times). The default values of the parameters before tuning, the searching thresholds, and the optimal value combination tuned by the MOPSO is illustrated in Table 1.

### 2.6. Performance Evaluation

The identification of UBPs is a typical binary classification problem that requires reliable evaluation processes and metrics. When training the model and tuning the parameters on the training dataset, we used 5-fold cross-validation to evaluate the model. First, the training dataset was randomly divided into five equal subsets. Then, one subset was chosen as the validation dataset, while all of the remaining samples were used for training. This process was repeated ten times to build ten submodels. Finally, the average validation results between the rounds were used as an estimate of the identifier. After training the model, the samples in the testing dataset were tested to assess the identification model’s ability to predict the first seen samples.

To quantitatively evaluate the proposed identifier, six measurements that are widely used for binary classification problems were adopted in this study: sensitivity (Sen), specificity (Spe), precision (Pre), accuracy (ACC), F1-measure (F1), and the Matthews correlation coefficient (MCC). Sen measures the proportion of observed UBPs that are correctly identified. Spe measures the proportion of observed non-UBPs that are correctly identified. Pre measures the proportion of predicted UBPs that are correctly identified. ACC measures the proportion of samples that are correctly identified. The F1-measure is a comprehensive measurement of test accuracy that considers both the Sen and the Pre. The value range of these five metrics is [0,1]. The higher the coefficient, the better the prediction performance. MCC is the correlation coefficient between the observed and predicted classification values, whose range is [−1,1]. The coefficient value 1 represents an entirely correct prediction, −1 represents a completely wrong prediction, and 0 represents a random prediction. MCC was regarded as the most reliable evaluation metric when tuning the model.

(5) $$Sen=\frac{TP}{TP+FN}$$

(6) $$Spe=\frac{TN}{TN+FP}$$

(7) $$Pre=\frac{TP}{TP+FP}$$

(8) $$ACC=\frac{TP+TN}{TP+TN+FP+FN}$$

(9) $$F1=2\times \frac{Sen\times Pre}{Sen+Pre}$$

(10) $$MCC=\frac{TP\times TN-FP\times FN}{\sqrt{\left(TP+FP\right)\times \left(TP+FN\right)\times \left(TN+FP\right)\times \left(TN+FN\right)}}$$

where TP, FP, TN, and FN represent true positive, false positive, true negative, and false negative, respectively.

## 3. Results and Discussion

### 3.1. Comparison of Different Classifiers

A powerful binary classifier is the foundation of a predictor. Thus, we tested six commonly used machine learning methods via cross-validation, including naïve Bayes (NB), multi-layer perceptron (MLP), support vector machine (SVM), adaptive boosting (AdaBoost), random forest (RF), and extreme gradient boosting (XGBoost). According to Table 2, XGBoost achieved the best prediction performance and was chosen as the classifier for the predictor. Notably, the last three methods were ensemble learning-based and significantly outperformed the others.

### 3.2. The Feature Selection Result

**Figure 1:**
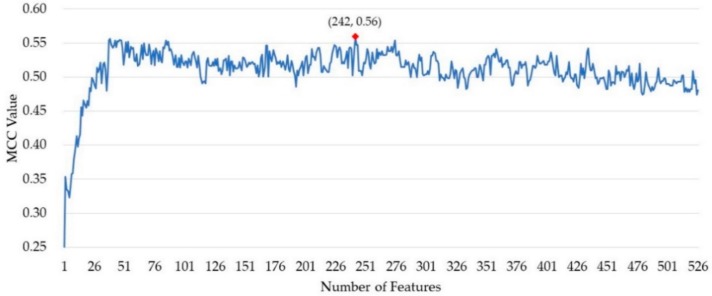
The Matthews correlation coefficient (MCC) value of the models in the process of incremental feature selection (IFS).

After ranking the importance of the features by random forest, 526 out of 820 features remained. The incremental feature selection (IFS) strategy was used to build a series of feature subset by increasing the features one by one. For each feature subset, a model was built and then evaluated over cross-validation. The performance of these prediction models was measured by the MCC value. As shown in Figure 1, the MCC value reached its maximum when 242 features were collected as the optimal feature subset. The predictor obtained an MCC value of 0.490 using all the features and obtained an MCC of 0.560 using the optimal feature subset.

**Figure 2:**
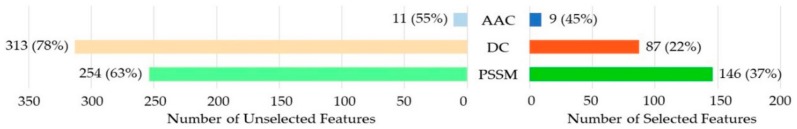
Distribution of each kind of feature in the optimal feature subset. AAC: amino acid composition; DC: dipeptide composition; PSSM: position-specific scoring matrix.

To discover the contributions of different types of features, we investigated the distribution of each kind of feature in the optimal feature subset. As shown in Figure 2, 9 out of 20 (45%) for the amino acid composition (AAC), 87 out of 400 (22%) for dipeptide composition (DC), and 146 out of the position-specific scoring matrix (PSSM) were selected for the optimal feature subset. The PSSM was obviously much better than the others. Although the number of DC in the optimal feature subset was higher than that of the AAC, the percentage of the selected DC among all the DCs was much lower than that of the AAC. We thus considered AAC to be more effective than DC.

### 3.3. The Result of Parameter Tuning

XGBoost is a highly sophisticated algorithm that involves multiple parameters. To leverage the advantages of XGBoost, we tuned the eight most common parameters using multi-objective particle swarm optimization (MOPSO). The prediction performance of the predictor before and after the parameter tuning over the cross-validation and independent validation are shown in Table 3. The prediction performance of the predictor was significantly improved after parameter tuning. This demonstrated that XGBoost was parameter-sensitive, and the process of parameter tuning was necessary.

### 3.4. Case Studies

We investigated four proteins that composed a mitochondrial respiratory complex II (PDB ID: 1YQ4 [40]) as case studies to demonstrate the effectiveness of UBPs-Pred. The UniProt IDs of the four proteins were Q9YHT1 (SdhA), Q9YHT2 (SdhB), D0VWW3 (SdhC), and Q5ZIS0 (SdhD). It should be noted that the sequences of these proteins were different in PDB and UniProt, and we used the version of UniProt. UBPs-Pred identified that SdhB, SdhC, and SdhD were ubiquinone-binding proteins, but SdhA was not a ubiquinone-binding protein. SdhB and SdhD were annotated as ubiquinone-binding proteins in UniProt. Although SdhC was not annotated as a UBP in UniProt, previous work demonstrated that SdhC contained ubiquinone binding sites [41]. These proteins are potential drug targets through which the nuclear SdhB, SdhC, and SdhD genes encoding complex II are considered to be tumor suppressor genes. Furthermore, in SdhC, mutation (Gly-713 to Glu) leads to the increased production of reactive oxygen species and premature aging that shortens the life span [42,43]. The sequence and structure information of these proteins can be found in the Supplementary Materials (Data S2: CaseStudies.zip).

**Figure 3:**
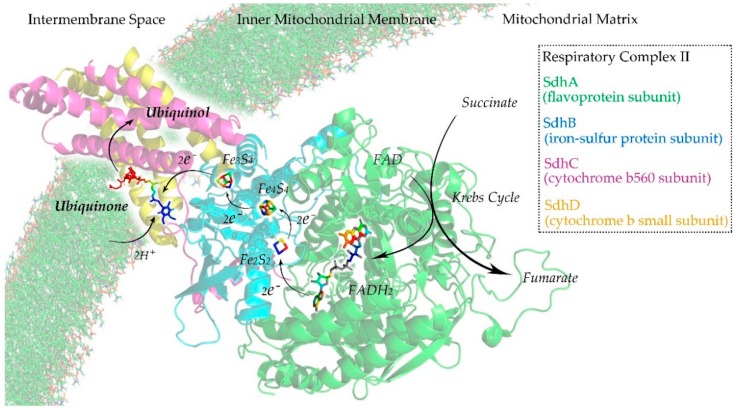
Illustration of the respiratory complex II of the mitochondrial respiratory chain.

Respiratory complex II or succinate dehydrogenase (Sdh) is an enzyme complex that can be found in the inner mitochondrial membrane. It is the only enzyme that participates in both the Krebs cycle [44] and the mitochondrial respiratory chain [45]. As illustrated in Figure 3, Sdh is composed of four subunits: a flavoprotein subunit (SdhA), an iron-sulfur protein subunit (SdhB), a cytochrome b560 subunit (SdhC), and a cytochrome b small subunit (SdhD) [46]. SdhA and SdhB are hydrophilic proteins where the enzymatic activity of the complex takes place. SdhC and SdhD are hydrophobic transmembrane proteins that anchor to the inner mitochondrial membrane. In the Krebs cycle, Sdh catalyzes the oxidation of succinate to fumarate with the reduction of ubiquinone to ubiquinol [47]. In the mitochondrial respiratory chain, the fully oxidized form of flavin adenine dinucleotide (FAD) is reduced to its hydroquinone form (FADH_2_). Electrons flow from FAD to FADH_2_ and are then transferred to ubiquinone through a series of iron–sulfur clusters: Fe_2_S_2_, Fe_4_S_3_, and Fe_3_S_4_. The ubiquinone undergoes reversible redox cycling between its oxidized form (ubiquinone) and its reduced form (ubiquinol). This redox cycling allows the ubiquinone to function as an electron carrier in the mitochondrial respiratory chain [48,49].

### 3.5. Ubiquinone-Binding Domain Analysis

**Figure 4:**
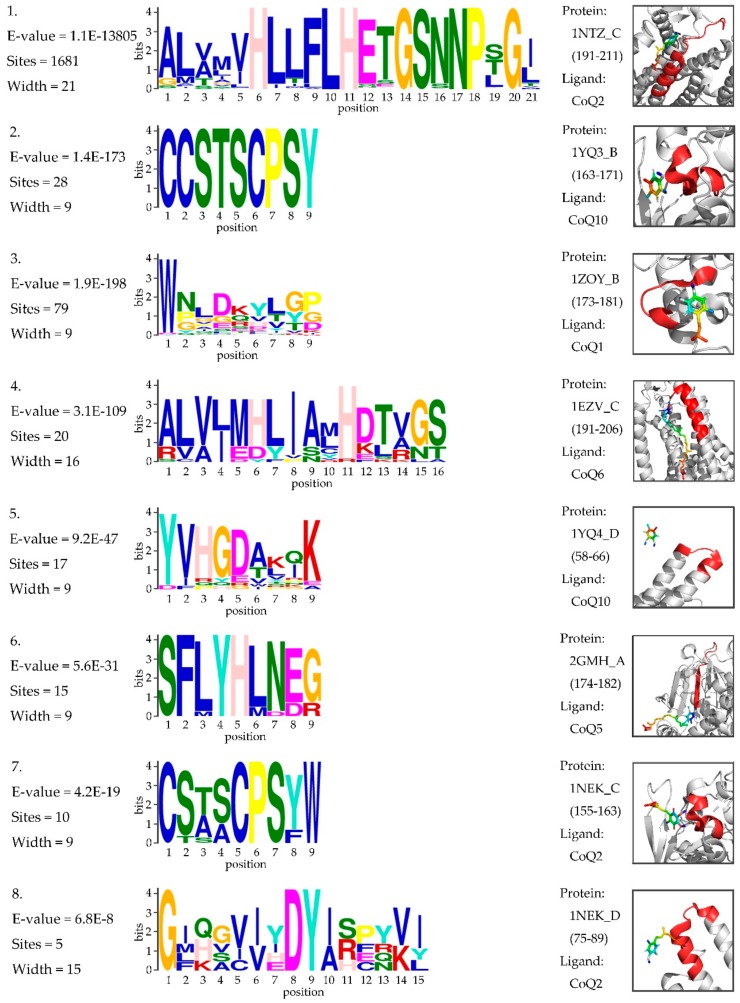
Sequence logos of the motif within the ubiquinone-binding domains. The threshold of the E-value is 0.05. “Sites” represents the number of sites contributing to the construction of the motif. “Width” represents the width of the motif. The 3D visualization on the right is an example of the corresponding motif. “Protein” represents the PDB ID_Chain (domain). “Ligand” represents the type of ubiquinone.

In genetics, a protein sequence motif is a sequence pattern that is widespread and has biological significance. We tried to determine the motif within ubiquinone-binding domains that might assist the discovery of potential drug targets. The ubiquinone-binding domains were extracted from all 10,224 UBPs in the Swiss-Prot database. A total of 1803 binding sites were annotated for 1791 UBPs. The ubiquinone-binding domain for a given binding site was constructed by slicing a sequence fragment with 21 residues for which the binding site was the center. We determined the motifs of these sequence fragments by using MEME [50]. The statistical significance of each motif was measured by its E-value. Motifs with an E-value within 0.05 were considered to be statistically significant, and eight motifs were identified. Figure 4 shows the sequence logos of eight motifs and the 3D visualizations of these examples. Details on motif discovery can be found in the Supplementary Materials (Data S3: BindingDomain.zip).

### 3.6. Distribution of UBPs

#### 3.6.1. Most UBPs are Membrane Proteins

As of June 5, 2019, a total of 10,224 UBPs were found in the Swiss-Prot database. According to the statistics, 8880 out of 10,224 proteins (86.9%) were membrane proteins (MPs). Among them, 6085 proteins (68.5%) were transmembrane proteins (TMPs). All of the TMPs were α-helical transmembrane proteins (α-TMPs). After preprocessing, we collected 524 UBPs to construct the benchmark datasets, including the training dataset and the testing dataset. According to the statistics of the selected UBPs, 387 out of 524 proteins (73.9%) were membrane proteins (MPs). Among them, 265 proteins (68.5%) were α-helical transmembrane proteins (α-TMPs). All of the TMPs were α-helical transmembrane proteins (α-TMPs). It was obvious that the majority of UBPs were membrane proteins. The statistics of UBP types can be found in Supplementary Materials (Distribution.zip).

#### 3.6.2. Superfamilies of UBPs

**Figure 5:**
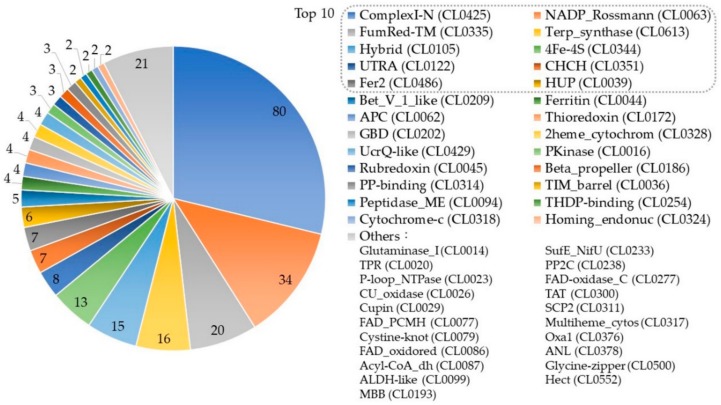
The superfamily distribution of the selected Ubiquinone-binding proteins (UBPs). The digital labels on the chart represent the number of UBPs that the superfamily contains. The names of the categories listed in the legend are the clan name in the Pfam database. All superfamilies in the “Others” category contain one protein.

The protein superfamily is the largest protein clade with common ancestry. This common ancestry is usually inferred from the similarity of proteins’ sequence, structure, and function. A superfamily typically contains several homologous protein families that have similar motifs or are involved in similar biological processes. We analyzed the superfamily distribution of the selected UBPs by extracting the clan annotation from the Pfam protein family database [51]. A total of 280 out of 525 UBPs were annotated by Pfam, and the statistical results are illustrated in Figure 5.

A total of 47 protein superfamilies appeared in the statistics, and the top 10 superfamilies contained about 74.1% UBPs. The top three superfamilies were ComplexI-N (CL0425), NADP_Rossmann (CL0063), and FumRed-TM (CL0335). ComplexI-N (CL0425) contained 80 UBPs. Proteins in this superfamily were part of respiratory complex I, which is associated with proton translocation across the membrane by catalyzing the electron transfers from NADH to ubiquinone. NADP_Rossmann (CL0063) [52] contained 34 UBPs and was a class of redox enzymes that contains two domains: one is a catalytic domain that confers the precise reaction of the enzyme, and the other one is the Rossmann domain that binds nicotinamide adenine dinucleotide (NAD) and FAD. FumRed-TM (CL0335) included 20 UBPs that contain transmembrane proteins from both the succinate dehydrogenase and fumarate reductase complexes [53]. Information about the superfamilies of UBPs can be found in the Supplementary Materials (Data S4: Distribution.zip).

UBPs appeared in various superfamilies that were involved in multiple complex biological processes. In order to understand the functions of UBPs, especially in the human body, we further analyzed the gene ontology enrichment and KEGG pathway enrichment of UBPs in the human species.

### 3.7. Gene Ontology Enrichment Analysis

Gene ontology (GO) is an important bioinformatics project that unifies information of genes and gene products across species. The basic information of GO contains information about the biological process (BP), cell component (CC), and molecular function (MF). The enrichment analysis of GO was used to test whether the queried set of genes was statistically enriched in a GO term, which could be measured by P-values:

(11) $$P-value=1-\overset{m-1}{\underset{i=0}{\sum }}\frac{\left(\begin{array}{c} M\\ i \end{array}\right)\left(\begin{array}{c} N-M\\ n-1 \end{array}\right)}{\left(\begin{array}{c} N\\ n \end{array}\right)}$$

where M is the number of all genes that are annotated by certain GO terms, m is the number of query genes annotated by certain GO terms, N is the number of all the genes of the specific organism that are annotated in GO, and n is the number of query genes annotated by the GO term. The cut-off for the P-value was set to 0.05.

**Figure 6:**
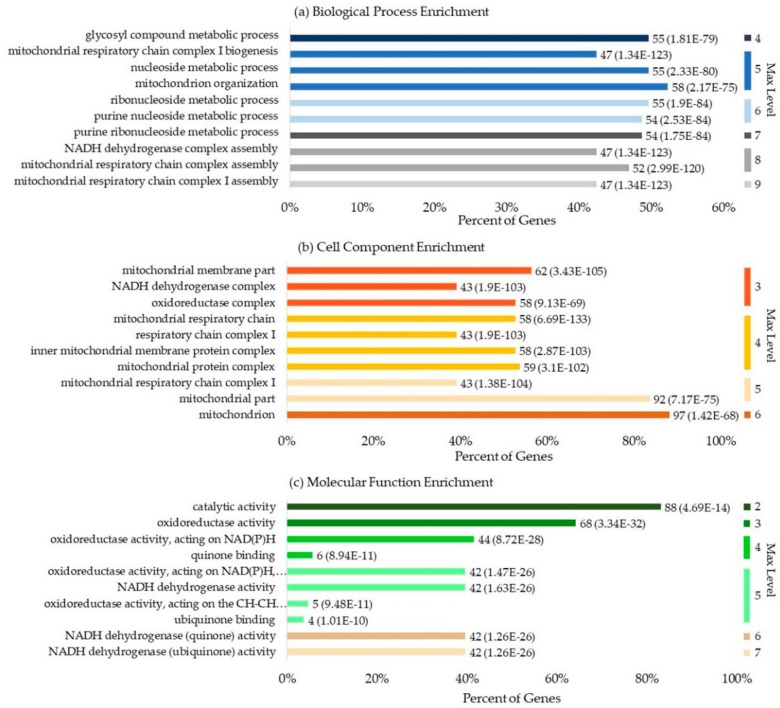
The general information of the gene ontology (GO) enrichment analysis result of human UBPs: (**a**) enriched biological processes; (**b**) enriched cell components; (**c**) enriched molecular functions. The description on the left side of the bar refers to the name of the gene term. “Percent of Genes” refers to the percentage of the number of genes involved in a given term compared to the total number of genes in the query proteins. The digital label on the right side of the bar of a gene term refers to the number of the genes involved in this term and its corresponding P-value. “Max Level” refers to the maximal annotated level of the given term in the GO graph. Different colors refer to the different max levels. Terms with the same max level are sorted according to P-value.

We obtained information regarding 113 human ubiquinone-binding proteins from Swiss-Prot. Figure 6 illustrates general information of the GO enrichment analysis results for these proteins, which feature 10 significantly enriched terms in BP, CC, and MF. A total of 2225 BPs were enriched, and this was considered statistically significant for 923 of them. The mitochondrial respiratory chain and metabolic processes were the most highly enriched biological processes. In total, 266 CCs were enriched, and this was considered statistically significant for 130 of them. The mitochondrial associated cell components were highly enriched; 407 MFs were enriched, and this was considered statistically significant for 140 of them. Apart from ubiquinone binding, catalytic activity, oxidoreductase activity, and dehydrogenase activity were the three molecular functions that were observed to be the most highly enriched.

### 3.8. KEGG Pathway Enrichment Analysis

The Kyoto Encyclopedia of Genes and Genomes (KEGG) is a bioinformatics database that unifies information on genomes, biological pathways, diseases, drugs, and chemical substances. Enrichment analysis of the KEGG pathway was used to test whether a query set of genes was statistically enriched in the KEGG pathway. This enrichment could be measured by the P-value using the formula used for GO enrichment analysis.

**Figure 7:**
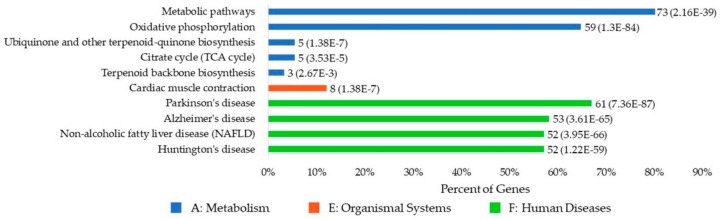
The top 10 significantly enriched KEGG (Kyoto Encyclopedia of Genes and Genomes) pathways of human UBPs. The description on the left side of the bar refers to the name of the KEGG pathway. “Percent of Genes” refers to the percentage of the number of genes involved in a given pathway compared to the total number of genes in the query proteins. The digital label on the right side of the bar of a gene term refers to the number of the genes involved in this pathway and the corresponding P-value. Different colors refer to the different categories of the pathways. Pathways of the same category are sorted by P-value.

Figure 7 illustrates the top 10 significantly enriched KEGG pathways for the human UBPs. For the 113 human UBPs, 70 pathways were enriched, and 11 of them were considered statistically significant. Three categories of pathways were enriched, including five metabolism pathways, one organismal system pathway, and four human disease pathways. Parkinson’s disease, Alzheimer’s disease, liver disease, and Huntington’s disease are the top four disease pathways associated with UBPs. Details about the KEGG pathway analysis can be found in the Supplementary Materials (Data S6: KEGG.zip).

## 4. Conclusions

In this study, we proposed the first UBPs predictor (UBPs-Pred), for which three types of sequence-derived features were collected: AAC, DC, and PSSM. Subsequently, a feature selection strategy that combined RF and IFS was used to build the optimal feature subset. Then, the selected features were fed into XGBoost, and the parameters of XGBoost were tuned using MOPSO. The experimental results demonstrated excellent prediction performance, with an MCC value of 0.517.

We then analyzed the UBPs using bioinformatics methods. By analyzing the ubiquinone-binding domains, we found eight motifs that were considered statistically significant. By analyzing the distribution of UBPs, we found that 86.9% of UBPs were membrane proteins. UBPs appeared in 47 superfamilies, and the top 10 superfamilies contained about 74.1% UBPs. ComplexI-N (CL0425), NADP_Rossmann (CL0063), and FumRed-TM (CL0335) were the top three superfamilies. By analyzing the GO enrichment of human UBPs, we found that 923 BP, 130 CC, and 140 MF were statistically significant. The mitochondrial respiratory chain and metabolic processes were the most strongly enriched biological processes. The mitochondrial associated cell components were highly enriched. Apart from ubiquinone binding, catalytic activity, oxidoreductase activity, and dehydrogenase activity were the three molecular functions that were found to be the most highly enriched. By analyzing the KEGG pathway enrichment of human UBPs, we found that 11 pathways were statistically significant. Among them, Parkinson’s disease, Alzheimer’s disease, liver disease, and Huntington’s disease were the top four disease pathways associated with UBPs.

## Figures and Tables

**Table 1:** Information about the parameters of XGBoost tuning by multi-objective particle swarm optimization (MOPSO) in this work: name, description, default value, threshold while searching, and tuned value.

Parameter	Description	Default	Threshold	Tuned
**Booster Parameters**				
learning_rate	Step size shrinkage	0.10	[0,0.5]	0.08
n_estimators	Number of trees	100	[100,2,000]	162
max_depth	The maximum depth of a tree	3	[1,10]	8
subsample	Percentage of samples used per tree	1.00	[0,1]	0.75
colsample_bytree	Percentage of features used per tree	1.00	[0,1]	0.12
**Learning Task Parameters**				
gamma	Controls a given node will split or not	0	[0,1]	0.83
reg_alpha	L1 regularization term on weight	0	[0,1]	0.08
reg_lambda	L2 regularization term on weights	1.00	[0,2]	

**Table 2:** Comparison of the different classifiers.

Classifier	Sen ^1^	Spe ^2^	Pre ^3^	ACC ^4^	F1 ^5^	MCC ^6^
NB	0.536	0.767	0.696	0.650	0.604	0.311
MLP	0.594	0.738	0.744	0.675	0.629	0.377
SVM	0.688	0.705	0.698	0.695	0.692	0.393
AdaBoost	0.704	0.734	0.723	0.719	0.712	0.438
RF	0.651	0.814 ^7^	0.781	0.734	0.708	0.474
XGBoost	0.754	0.759	0.756	0.755	0.753	0.511

^1–6^ are the performance evaluation indicators of the predictor: Sen represents the sensitivity; Spe represents the specificity; Pre represents the precision; ACC represents the accuracy; F1 represents the F1-measure; MCC represents the Matthews correlation coefficient (MCC). ^7^ The bolded parts represent the highest value of the corresponding evaluation indicator.

**Table 3:** Comparison of the prediction performance before and after parameter tuning through cross-validation and independent validation.

Models	Sen	Spe	Pre	ACC	F1	MCC
**Cross-Validation**						
Default parameters	0.759	0.786	0.779	0.772	0.768	0.545
Tuned parameters	**0.746** *	**0.807**	**0.796**	**0.776**	**0.768**	**0.577**
**Independent Validation**						
Default parameters	0.649	0.760	0.727	0.705	0.686	0.411
Tuned parameters	**0.703**	**0.811**	**0.788**	**0.757**	**0.743**	**0.517**

* The bolded parts represent the highest value of the corresponding evaluation indicator.

## Supplementary Materials

The following are available online at https://www.mdpi.com/2073-4409/9/2/520/s1, Data S1: BenchmarkDatasets.zip, Data S2: CaseStudies.zip, Data S3: BindingDomain.zip, Data S4: Distribution.zip, Data S5: GeneOntology.zip, Data S6: KEGG.zip.

## References

[1] Ernster L., Dallner G. (1995). Biochemical, physiological and medical aspects of ubiquinone function. Biochim. Biophys. Acta.

[2] Wang Y., Hekimi S. (2016). Understanding Ubiquinone. Trends Cell Biol..

[3] Crane F.L. (2001). Biochemical functions of coenzyme Q10. J. Am. Coll. Nutr..

[4] Jafari M., Mousavi S.M., Asgharzadeh A., Yazdani N. (2018). Coenzyme Q10 in the treatment of heart failure: A systematic review of systematic reviews. Indian Heart J..

[5] Sobirin M.A., Herry Y., Sofia S.N., Uddin I., Rifqi S., Tsutsui H. (2019). Effects of coenzyme Q10 supplementation on diastolic function in patients with heart failure with preserved ejection fraction. Drug Discov. Ther..

[6] Zhang X., Shi Z., Liu Q., Quan H., Cheng X. (2019). Effects of coenzyme Q10 intervention on diabetic kidney disease: A systematic review and meta-analysis. Medicine.

[7] Xu Y., Liu J., Han E., Wang Y., Gao J. (2019). Efficacy of coenzyme Q10 in patients with chronic kidney disease: Protocol for a systematic review. BMJ Open.

[8] Tafazoli A. (2017). Coenzyme Q10 in breast cancer care. Future Oncol..

[9] Vetvicka V., Vetvickova J. (2018). Combination Therapy with Glucan and Coenzyme Q10 in Murine Experimental Autoimmune Disease and Cancer. Anticancer Res..

[10] Tuz K., Li C., Fang X., Raba D.A., Liang P.D., Minh D.D.L., Juarez O. (2017). Identification of the Catalytic Ubiquinone-binding Site of Vibrio cholerae Sodium-dependent NADH Dehydrogenase A NOVEL UBIQUINONE-BINDING MOTIF. J. Biol. Chem..

[11] Jenkins B.J., Daly T.M., Morrisey J.M., Mather M.W., Vaidya A.B., Bergman L.W. (2016). Characterization of a Plasmodium falciparum Orthologue of the Yeast Ubiquinone-Binding Protein, Coq10p. PLoS ONE.

[12] Fathima A.J., Murugaboopathi G., Selvam P. (2018). Pharmacophore Mapping of Ligand Based Virtual Screening, Molecular Docking and Molecular Dynamic Simulation Studies for Finding Potent NS2B/NS3 Protease Inhibitors as Potential Anti-dengue Drug Compounds. Curr. Bioinform..

[13] Basith S., Manavalan B., Shin T.H., Lee G. (2018). iGHBP: Computational identification of growth hormone binding proteins from sequences using extremely randomised tree. Comput. Struct. Biotec. J..

[14] Chauhan S.C.T., Ahmad S.D.R. (2019). Enabling full-length evolutionary profiles based deep convolutional neural network for predicting DNA-binding proteins from sequence. Proteins.

[15] Pan X.Y., Rijnbeek P., Yan J.C., Shen H.B. (2018). Prediction of RNA-protein sequence and structure binding preferences using deep convolutional and recurrent neural networks. BMC Genomics.

[16] UniProt C. (2019). UniProt: A worldwide hub of protein knowledge. Nucleic Acids Res..

[17] Zou Q., Lin G., Jiang X., Liu X., Zeng X. (2018). Sequence clustering in bioinformatics: An empirical study. Brief. Bioinform..

[18] Huang Y., Niu B., Gao Y., Fu L., Li W. (2010). CD-HIT Suite: A web server for clustering and comparing biological sequences. Bioinformatics.

[19] Zhang J., Chai H.T., Guo S., Guo H.P., Li Y.L. (2018). High-Throughput Identification of Mammalian Secreted Proteins Using Species-Specific Scheme and Application to Human Proteome. Molecules.

[20] Zhang J., Chai H., Gao B., Yang G., Ma Z. (2018). HEMEsPred: Structure-Based Ligand-Specific Heme Binding Residues Prediction by Using Fast-Adaptive Ensemble Learning Scheme. IEEE/ACM Trans. Comput. Biol. Bioinform..

[21] Khan M., Hayat M., Khan S.A., Iqbal N. (2017). Unb-DPC: Identify mycobacterial membrane protein types by incorporating un-biased dipeptide composition into Chou’s general PseAAC. J. Theor. Biol..

[22] Zhang J., Chai H.T., Yang G.F., Ma Z.Q. (2017). Prediction of bioluminescent proteins by using sequence-derived features and lineage-specific scheme. BMC Bioinform..

[23] Jeong J.C., Lin X., Chen X.W. (2011). On position-specific scoring matrix for protein function prediction. IEEE/ACM Trans. Comput. Biol. Bioinform..

[24] Zeng B., Honigschmid P., Frishman D. (2019). Residue co-evolution helps predict interaction sites in alpha-helical membrane proteins. J. Struct. Biol..

[25] Zangooei M.H., Jalili S. (2012). Protein secondary structure prediction using DWKF based on SVR-NSGAII. Neurocomputing.

[26] Qu K.Y., Wei L.Y., Zou Q. (2019). A Review of DNA-binding Proteins Prediction Methods. Curr. Bioinform..

[27] Wei L., Tang J., Zou Q. (2017). Local-DPP: An improved DNA-binding protein prediction method by exploring local evolutionary information. Inform. Sci..

[28] Altschul S.F., Madden T.L., Schaffer A.A., Zhang J., Zhang Z., Miller W., Lipman D.J. (1997). Gapped BLAST and PSI-BLAST: A new generation of protein database search programs. Nucleic Acids Res..

[29] Ru X., Li L., Zou Q. (2019). Incorporating Distance-Based Top-n-gram and Random Forest To Identify Electron Transport Proteins. J. Proteome Res..

[30] Lv Z., Jin S., Ding H., Zou Q. (2019). A Random Forest Sub-Golgi Protein Classifier Optimized via Dipeptide and Amino Acid Composition Features. Front. Bioeng. Biotechnol..

[31] Breiman L. (2001). Random Forests. Machine Learning.

[32] Zhu R.Q., Zeng D.L., Kosorok M.R. (2015). Reinforcement Learning Trees. J. Am. Stat. Assoc..

[33] Liu H., Setiono R. (1998). Incremental feature selection. Appl. Intell..

[34] Chen T.Q., Guestrin C. (2016). XGBoost: A Scalable Tree Boosting System. Kdd’16: Proceedings of the 22nd Acm Sigkdd International Conference on Knowledge Discovery and Data Mining.

[35] Li W., Yin Y., Quan X., Zhang H. (2019). Gene Expression Value Prediction Based on XGBoost Algorithm. Front. Genet..

[36] Pang L., Wang J., Zhao L., Wang C., Zhan H. (2018). A Novel Protein Subcellular Localization Method With CNN-XGBoost Model for Alzheimer’s Disease. Front. Genet..

[37] Wang J., Gribskov M. (2019). IRESpy: An XGBoost model for prediction of internal ribosome entry sites. BMC Bioinform..

[38] Coello C.C., Lechuga M.S. MOPSO: A proposal for multiple objective particle swarm optimization. Proceedings of the 2002 Congress on Evolutionary Computation. CEC’02 (Cat. No. 02TH8600).

[39] Eberhart R., Kennedy J. (1995). Particle swarm optimization. Proc. IEEE Int. Conf. Neural Netw..

[40] Huang L.S., Sun G., Cobessi D., Wang A.C., Shen J.T., Tung E.Y., Anderson V.E., Berry E.A. (2006). 3-Nitropropionic acid is a suicide inhibitor of mitochondrial respiration that, upon oxidation by Complex II, forms a covalent adduct with a catalytic base arginine in the active site of the enzyme. J. Biol Chem.

[41] Horsefield R., Yankovskaya V., Sexton G., Whittingham W., Shiomi K., Omura S., Byrne B., Cecchini G., Iwata S. (2006). Structural and computational analysis of the quinone-binding site of complex II (succinate-ubiquinone oxidoreductase): A mechanism of electron transfer and proton conduction during ubiquinone reduction. J. Biol Chem.

[42] Ishii N., Fujii M., Hartman P.S., Tsuda M., Yasuda K., Senoo-Matsuda N., Yanase S., Ayusawa D., Suzuki K. (1998). A mutation in succinate dehydrogenase cytochrome b causes oxidative stress and ageing in nematodes. Nature.

[43] Ishii T., Yasuda K., Akatsuka A., Hino O., Hartman P.S., Ishii N. (2005). A mutation in the SDHC gene of complex II increases oxidative stress, resulting in apoptosis and tumorigenesis. Cancer Res..

[44] Krebs H.A. (1940). The citric acid cycle and the Szent-Gyorgyi cycle in pigeon breast muscle. Biochem. J..

[45] Oyedotun K.S., Lemire B.D. (2004). The quaternary structure of the Saccharomyces cerevisiae succinate dehydrogenase. Homology modeling, cofactor docking, and molecular dynamics simulation studies. J. Biol. Chem..

[46] Sun F., Huo X., Zhai Y., Wang A., Xu J., Su D., Bartlam M., Rao Z. (2005). Crystal structure of mitochondrial respiratory membrane protein complex II. Cell.

[47] Yankovskaya V., Horsefield R., Tornroth S., Luna-Chavez C., Miyoshi H., Leger C., Byrne B., Cecchini G., Iwata S. (2003). Architecture of succinate dehydrogenase and reactive oxygen species generation. Science.

[48] Schneider H., Lemasters J.J., Hackenbrock C.R. (1982). Lateral diffusion of ubiquinone during electron transfer in phospholipid- and ubiquinone-enriched mitochondrial membranes. J. Biol. Chem..

[49] Aberg F., Appelkvist E.L., Dallner G., Ernster L. (1992). Distribution and redox state of ubiquinones in rat and human tissues. Arch. Biochem. Biophys..

[50] Bailey T.L., Boden M., Buske F.A., Frith M., Grant C.E., Clementi L., Ren J., Li W.W., Noble W.S. (2009). MEME SUITE: Tools for motif discovery and searching. Nucleic Acids Res..

[51] El-Gebali S., Mistry J., Bateman A., Eddy S.R., Luciani A., Potter S.C., Qureshi M., Richardson L.J., Salazar G.A., Smart A. (2019). The Pfam protein families database in 2019. Nucleic Acids Res..

[52] Bashton M., Chothia C. (2002). The geometry of domain combination in proteins. J. Mol. Biol.

[53] Lancaster C.R.D., Kroger A., Auer M., Michel H. (1999). Structure of fumarate reductase from Wolinella succinogenes at 2.2 angstrom resolution. Nature.

