# Supplementary material for: Computational Identification and Analysis of Ubiquinone-Binding Proteins
Source: Cells. 2020 Feb 24;9(2):520. doi: 10.3390/cells9020520 (PMC7072731; doi:10.3390/cells9020520)
Supplement: Supplementary file 1 [file cells-09-00520-s001.zip › SupplementaryMaterials/3.BindingDomain.zip!/BindingDomainAnalysis/ResultsFromMEME/ResultsPDF_in_PDF_Format.pdf]

MEME

Multiple Em for Motif Elicitation

For further information on how to interpret these results please access http://meme-suite.org/.
To get a copy of the MEME software please access http://meme-suite.org.

If you use MEME in your research, please cite the following paper:
Timothy L. Bailey and Charles Elkan, "Fitting a mixture model by expectation maximization to discover motifs in biopolymers", *Proceedings of the Second International Conference on Intelligent Systems for Molecular Biology*, pp. 28-36, AAAI Press, Menlo Park, California, 1994. [pdf]

Discovered Motifs | Motif Locations | Inputs & Settings | Program Information | Results in Text Format NEW | Results in XML Format NEW

Discovered Motifs

	Logo	E-value	Sites	Width	More	Submit/D
1.	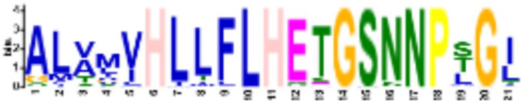	1.1e-13805	1681	21	↧	...
2.	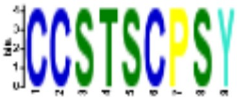	1.4e-173	28	9	↧	...
3.	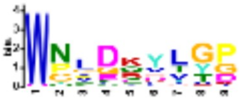	1.9e-198	79	9	↧	...
4.	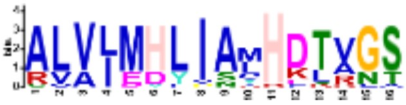	3.1e-109	20	16	↧	...
5.	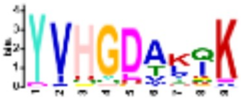	9.2e-047	17	9	↧	...
6.	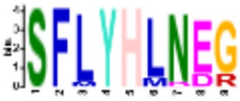	5.6e-031	15	9	↧	...
7.	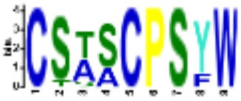	4.2e-019	10	9	↧	...
8.	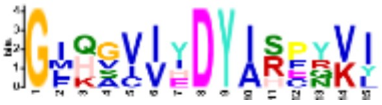	6.8e-008	5	15	↧	...
9.	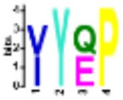	2.8e-003	2	4	↧	...
10.	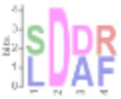	2.1e-001	2	4	↧	...

Stopped because requested number of motifs (10) found.

Motif Locations

Output of sites suppressed because there were more than 1000 (primary) sequences.

Inputs & Settings

Sequences

Role	Source	Alphabet	Sequence Count	Total Size
Primary Sequences	SiteResult.fasta	Protein	1803	37863

Background Model

Source: built from the (primary) sequences

Order: 0

	Name	Freq.	Bg.
A	Alanine	0.0644	0.0644
C	Cysteine	0.00378	0.00378
D	Aspartic acid	0.00383	0.00383
E	Glutamic acid	0.0446	0.0446
F	Phenylalanine	0.045	0.045
G	Glycine	0.104	0.104
H	Histidine	0.0929	0.0929
I	Isoleucine	0.0493	0.0493
K	Lysine	0.00233	0.00233
L	Leucine	0.201	0.201
M	Methionine	0.03	0.03
N	Asparagine	0.0886	0.0886
P	Proline	0.0481	0.0481
Q	Glutamine	0.00256	0.00256
R	Arginine	0.00145	0.00145
S	Serine	0.0794	0.0794
T	Threonine	0.0724	0.0724
V	Valine	0.0575	0.0575
W	Tryptophan	0.00349	0.00349
Y	Tyrosine	0.00507	0.00507

Other Settings

**Motif Site Distribution**	ZOOPS: Zero or one site per sequence
**Objective Function**	E-value of product of p-values
**Starting Point Function**	E-value of product of p-values
**Site Strand Handling**	This alphabet only has one strand
**Maximum Number of Motifs**	10
**Motif E-value Threshold**	no limit
**Minimum Motif Width**	3
**Maximum Motif Width**	21
**Minimum Sites per Motif**	2
**Maximum Sites per Motif**	1803

Show Advanced Settings

MEME version

5.1.0 (Release date: Fri Oct 11 15:53:28 2019 -0700)

Reference

Timothy L. Bailey and Charles Elkan, "Fitting a mixture model by expectation maximization to discover motifs in biopolymers", *Proceedings of the Second International Conference on Intelligent Systems for Molecular Biology*, pp. 28-36, AAAI Press, Menlo Park, California, 1994.

Command line

```
meme SiteResult.fasta -protein -oc . -nostatus -time 18000 -mod zoops -nmotifs 10
-minw 3 -maxw 50 -objfun classic -markov_order 0
```
